# Metformin as an anti-cancer agent against bladder cancer acts via PD-L1 downregulation in an orthotopic mouse model

**DOI:** 10.1186/s12885-025-14930-2

**Published:** 2025-10-08

**Authors:** Chang-Ching Yeh, Pei-Chu Tsai, Yung-Deng Song, Yong-Seng Low, Chih-Hao Hsu, Shiu-Ju Yang, Yih-Yuan Chen, Horng-Yunn Dou

**Affiliations:** 1 National Institute of Infectious Diseases and Vaccinology, National Health Research Institutes, Zhunan, Miaoli Taiwan; 2 Department of Biochemical Science and Technology, National Chiayi University, Chia-Yi, Taiwan

**Keywords:** PD-L1, Immune checkpoint, Metformin, Type 2 diabetes, Bladder cancer

## Abstract

**Background:**

PD-L1 is a crucial immune checkpoint protein that limits the effectiveness of antitumor immunity. Blocking the PD-L1/PD-1 pathway has shown promise in cancer treatment, but low response rates to checkpoint inhibitors highlight the need for alternative therapeutic strategies. Metformin, a drug primarily used for diabetes, has gained attention as a potential cancer treatment due to its effects on PD-L1 expression, the tumor microenvironment, and its ability to inhibit the proliferation of tumor cells. Meta-analyses suggest that metformin may reduce the incidence and improve the prognosis of bladder cancer in patients with type 2 diabetes, but the exact mechanisms by which it exerts its effects remain unclear.

**Materials:**

This study employed a syngeneic orthotopic bladder cancer model with immunocompetent C57BL/6 mice to evaluate metformin’s therapeutic effects. Safe dosage was established through a maximum tolerated dose (MTD) test, identifying 150 mg/kg/day as suitable. Tumor size, body weight, survival, and PD-L1 expression were measured, while a tetrazolium-based assay assessed bladder cancer cell proliferation *in vitro*.

**Results:**

Findings revealed elevated PD-L1 gene and protein expression in mouse bladder cancer tissues. *In vitro*, metformin inhibited PD-L1 expression and proliferation of MB49 mouse bladder cancer cells. *In vivo*, metformin reduced cancer cachexia, tumor size, and improved survival at 150 mg/kg/day. Importantly, metformin attenuated tumor-induced PD-L1 upregulation in a dose-dependent manner.

**Conclusion:**

Overall, this study suggests that bladder tumors can increase PD-L1 expression to promote PD-L1–mediated intrinsic tumor growth pathways, and metformin effectively downregulates PD-L1 expression, suppresses bladder cancer cell proliferation, and prolongs survival in an orthotopic bladder cancer mouse model.

**Supplementary Information:**

The online version contains supplementary material available at 10.1186/s12885-025-14930-2.

## Background

Bladder cancer is the most prevalent malignancy of the urinary tract [1]. The incidence of bladder cancer is nearly three times higher in more developed countries than in developing countries and up to 75% of patients are diagnosed with non-muscle invasive bladder cancer (NMIBC), while the remaining 25% are diagnosed with muscle-invasive bladder cancer (MIBC) [2]. Currently, the standard treatment for NMIBC remains transurethral resection of the bladder tumor (TURBT) combined with intravesical delivery of Bacillus Calmette–Guérin (BCG) to prevent recurrence and progression [3]. However, patients may be unable to undergo induction and maintenance intravesical BCG immunotherapy due to intolerance [4] or issues with BCG availability [5]. Additionally, these therapies are associated with a high recurrence rate, and the risk of disease progression remains significant owing to the immunosuppressive microenvironment of bladder cancer [6]. The 5-year recurrence rates of NMIBC can exceed 50%, depending on tumor stage and grade [2, 7]. Additionally, NMIBC may progress to MIBC in up to 40% of cases following repeated recurrences, even after initial successful treatment. Patients with MIBC generally have a poor prognosis, with a 5-year overall survival rate of less than 50%, and no significant improvements in outcomes have been observed in recent decades [2].

Programmed death protein 1 (PD-1) is a transmembrane receptor expressed on tumor-infiltrating immune cells, including T cells, B cells, natural killer cells, macrophages and dendritic cell subsets [8]. Its ligand, programmed death ligand 1 (PD-L1), is frequently overexpressed on the surface of tumor cells across various cancer types, where it interacts with the PD-1 receptor [9]. This binding suppresses T cell function and negatively regulates the adaptive antitumor immune response [10, 11], thereby allowing tumor cells to evade immune surveillance [12]. Therapeutic agents that disrupt the PD-L1/PD-1 interaction can restore T cell cytotoxicity, thereby enhancing the immune system’s ability to target and eliminate tumors [13]. Accordingly, high levels of PD-L1 expression, as assessed by immunohistochemistry, may indicate more aggressive bladder tumors [14]. This is further supported by its association with advanced pathological stage at the time of resection [15] and higher mortality rates [16].

Therefore, therapeutic agents targeting the PD-L1/PD-1 pathway have attracted the attention of researchers for further investigation. Currently, the most commonly used approach involves the administration of targeted monoclonal antibodies that block inhibitory immune checkpoint molecules, thereby promoting T cell activation [17]. The efficacy of PD-1/PD-L1 immune checkpoint inhibitors (ICIs) in metastatic disease has prompted their investigation as a potential treatment for patients with high-risk NMIBC and MIBC. However, the low durable response rate (approximately 30%), systemic autoimmune adverse effects, and high costs associated with ICI immunotherapy remain significant challenges [18–21]. Therefore, there is an urgent need to identify effective strategies to enhance antitumor effects and reduce toxicity in order to prevent the progression and recurrence of NMIBC, as well as to improve survival rates in MIBC.

Metformin, a widely utilized first-line oral hypoglycemic agent for the management of type 2 diabetes, has been shown to be associated with decreased cancer risk and improved cancer-related mortality in patients with diabetes in various cancers [22, 23], including bladder cancer [24, 25]. Notably, recent studies reported that metformin showed the ability to reverse tumor immunosuppression either through the degradation or inhibition of PD-L1, thereby stimulating tumor-infiltrated immune cells and suppressing cancer cell growth in certain cancers [26–28]. Furthermore, retrospective studies have indicated that metformin may offer protective benefits against recurrence in patients with NMIBC and reduce cancer-specific mortality in individuals with MIBC [29, 30]. Although epidemiological studies suggest potential therapeutic benefits, the impact of metformin on PD-L1 expression in bladder cancer has yet to be investigated in preclinical animal models. The aim of this study was to assess the effects of metformin on bladder cancer using both *in vitro* and *in vivo* mouse orthotopic bladder cancer models, and to investigate the role of metformin in influencing PD-L1 expression.

## Methods

### Mice and intravesical treatment

7–12-week-old female wild-type C57BL/6J mice (National Laboratory Animal Center, Taiwan) were used for all animal experiments. Mice were randomly assigned to experimental groups and fed normal chow. They were housed under standard 12-hour light-dark (LD) cycles in a specific pathogen-free laboratory animal center at the National Health Research Institutes, Taiwan, which is accredited by AAALAC. All experimental procedures were performed in accordance with NIH guidelines (Guide for the Care and Use of Laboratory Animals) and approved by the Institutional Animal Care and Use Committee of the National Health Research Institutes, Taiwan (approval IDs: #NHRI-IACUC-109153-A-S03 and #NHRI-IACUC-110112-A-S02). To assess the antitumor effect of metformin (D150959, Sigma-Aldrich), we used the immunocompetent C57BL/6 mouse strain to establish an orthotopic bladder cancer animal model. C57BL/6J mice were subjected to water deprivation for 2 to 4 h before the experiment to prevent micturition during the intravesical treatment. After water deprivation, the mice were anesthetized with pentobarbital (80 mg/kg). Prior to intravesical tumor implantation, we created lesion sites in the bladder by using a urethral catheter (NIC-24G×3/4, NIPRO) and syringe (SS-01T, TERUMO) to aspirate an approximately 0.3-ml volume of the mucosal/glycosaminoglycan layer (6 times) to disrupt the urothelial cells of the bladder wall, followed by application of poly L-lysine (PLL) (P4707, Sigma-Aldrich) on the lesion sites for 20 min to provide positive Charges for tumor cell attachment and embedding. Following bladder pretreatment with PLL, 6× 10⁴ MB49 bladder cancer cells or DMEM (vehicle) were intravesically implanted into the bladder again using a catheter and syringe as above, and the catheter/syringe assembly was left in place for 1 h to prevent the injected tumor cells from flowing out of the bladder. In the course of intravesical administration, urethral catheters were lubricated to alleviate pain associated with transurethral catheterization. During the experiment, animals were observed daily to monitored pain or distress. Humane euthanasia was performed when necessary, including cases where body weight loss exceeded 20%, dehydration occurred, abnormal posture or positioning was observed (e.g., head-pressing, hunched back), or movement decreased. Mice reaching the Humane endpoint criteria or the experimental endpoint were euthanized using 100% CO₂ exposure via inhalation for over 5 min, with a gas displacement rate of 30–70% of the chamber volume per minute.

To assess the antitumor effects of metformin, C57BL/6 mice were randomly allocated to the following experimental groups: Vehicle, MB49 + phosphate-buffered saline (PBS), MB49 + 100 mg/kg metformin, and MB49 + 150 mg/kg metformin. Following tumor implantation, the respective doses of metformin were administered via intraperitoneal injection the following day and continued once daily for 28 days. At the humane or experimental endpoint (Day 29), the treated mice were euthanized and the bladder collected. Survival was monitored daily throughout the study. Body weight measurements were recorded on Day 0 and subsequently three times per week after the first intraperitoneal injection, continuing until the study endpoint (4 weeks) or a humane endpoint. At the experimental endpoint, or upon reaching the humane endpoint, the mice were weighed, euthanized, and their bladders collected and weighed. Cancer cachexia was evaluated using the delta body weight change ratio, which was calculated by subtracting the initial body weight (on Day 0) of the tumor group from the final body weight at the endpoint (excluding bladder/tumor weight). This value was then divided by the difference between the final body weight of healthy mice (excluding bladder weight) and their initial body weight on Day 0. This ratio was used to quantify cancer cachexia induced by MB49 tumor cells.

### Maximum tolerated dose test

For the maximum tolerated dose (MTD) of metformin, C57BL/6J mice were intraperitoneally injected with PBS (control), 150 mg/kg, 200 mg/kg, and 350 mg/kg of metformin daily for 28 days. Survival was monitored daily until Day 28. The body weight of the treated mice was recorded on Day 0 and subsequently three times a week for 4 weeks, following the first intraperitoneal injection on Day 1. The percentage change in body weight (%) was calculated by dividing the body weight on Day n by the body weight on Day 0 for each mouse and multiplying the result by 100. The data are presented as the percentage change in body weight (%).

### Immunohistochemistry

For histological analysis, the mice were perfused with saline followed by 10% formaldehyde in PBS, and the bladders were then collected. The bladder tissues were fixed in 10% formaldehyde/PBS for 24 h. The tissues were subsequently dehydrated using a graded series of ethanol, and embedded in paraffin. Tissue blocks were sectioned into 4-µm-thick slices. Prior to assessing PD-L1 protein expression by immunohistochemistry, the bladder sections were deparaffinized with xylene and rehydrated through a graded alcohol series. Antigen retrieval was performed by heating the sections on slides in an EDTA antigen retrieval buffer (pH 8; Trilogy, Cell Marque Corporation) using an electric pressure cooker (BSB 7008, Bio SB) for 15 min. Sections were blocked sequentially with 3% hydrogen peroxide for 20 min and then with 5% bovine serum albumin (BSA) in PBS + 0.1% Tween-20 (PBST) for an additional 30 min. PD-L1 (17952-1-AP, Proteintech) antibodies were diluted in PBST. The sections were incubated with the primary antibody at 4 °C overnight, followed by washing with PBST. The sections were then incubated with horseradish peroxidase (HRP)-labeled polymer (Dako) for 60 min and washed three times with PBST. Protein expression was visualized using the DAB Chromogen system (Dako). To validate the specificity of the primary antibody, a negative control was included, where the primary antibody was replaced with an isotype control antibody. The slides were counterstained with hematoxylin. The immunopositive areas in the bladder tissue sections were quantified using ImageJ software, and the signal values were expressed as the percentage of the positive area relative to the total tissue area.

### Cell cultures and treatments

The human bladder urothelial carcinoma cell lines T24 and 5637 were obtained from the Bioresource Collection and Research Center (BCRC, Hsinchu, Taiwan) and cultured in McCoy’s 5 A and RPMI-1640 media, respectively, each supplemented with 10% fetal bovine serum (FBS), penicillin (100 U/mL), and streptomycin (100 µg/mL). The mouse urothelial carcinoma cell line MB49 was purchased from Millipore and cultured in DMEM medium supplemented with 10% FBS, penicillin (100 U/mL), and streptomycin (100 µg/mL), and cells of passages 5–10 were used for the animal experiments. The MB49 tumor cells were grown to ~ 80% confluence and detached by 0.25% trypsin-EDTA. After neutralization with DMEM supplemented with 10% FBS, the cells were washed with DMEM without FBS thrice to avoid activating the mouse immune response to FBS during intravesical implantation of the tumor cells. For RNA extraction and immunoblot analysis, MB49 tumor cells were seeded at a density of 6 × 10⁵ cells per well in 6-well plates. The medium was removed the following day and replaced with growth medium containing varying concentrations of metformin for the specified duration.

### RNA extraction and QPCR analysis

Total RNA of metformin-treated cells and mouse bladder tumor tissue was extracted by Trizol reagent (Thermo Fisher Scientific, USA). cDNAs were synthesized from 1 µg total RNA using a RevertAid H Minus First Strand cDNA Synthesis Kit (#K1631, ThermoFisher). The mRNA levels of PD-L1 and GAPDH were detected by real-time quantitative PCR analysis using the QuantStudio 6 Flex system (Applied Biosystems, USA). PD-L1 and GAPDH levels were calculated relative to amounts found in a control sample, and the gene level was corrected for GAPDH mRNA levels to normalize for RNA input. Relative expression was quantified using the ΔΔCt method. Primers used for RT-PCR were as follows: *PD-L1* Fwd: 5′-AAGTCAATGCCCCATACCG-3′ and Rev: 5′-ACACTTCTCTTCCCACTCACG-3′; and *GAPDH* Fwd: 5′-CAAGGTCATCCATGACAACTTTG-3′ and Rev: 5′-GTCCACCACCCTGTTGCTGTAG-3′.

### Western blot analysis

Cellular proteins extracted from metformin-treated cells by RIPA buffer were resolved with 4–12% SDS-PAGE and transferred to polyvinylidene difluoride (PVDF) membranes. After transfer, PVDF membranes were blocked and blotted with PD-L1 (GTX31308, GeneTex), and GAPDH (ab8245, Abcam) antibodies at 4 °C overnight. Next, the membranes were washed and blotted with HRP-conjugated goat anti-rabbit antibody (111-035-003, Jackson laboratory) for PD-L1 and HRP-conjugated goat anti-mouse antibody (115-035-003, Jackson laboratory) for GAPDH at room temperature for 1 h. The membranes were washed and developed by using enhanced chemiluminescence substrates (Thermo) and exposed to film to detect the specific protein expression.

### Cell viability assay

Cell viability was assessed using the CellTiter 96^®^ AQueous One Solution assay (G3580, Promega). Cells were plated in 96-well plates at the following densities: MB49 at 2 × 10⁴ cells per well, T24 at 2.5 × 10³ cells per well, and 5637 at 5 × 10³ cells per well. The following day, the medium was replaced with fresh growth medium containing metformin at varying concentrations for the indicated time. Cell viability was measured by evaluating the reduction of soluble formazan by mitochondrial dehydrogenases in viable cells, and absorbance was read at 490 nm. The experiments were performed independently in triplicate.

### Statistical analysis

Statistical analyses were conducted using GraphPad Prism version 8 (GraphPad Software Inc., San Diego, CA, USA). Data are expressed as means ± SEM. To assess differences between the vehicle and treatment groups, a two-tailed t-test was performed. For comparisons involving multiple groups, one-way ANOVA followed by post hoc tests (Fisher’s Least Significant Difference (LSD) or Dunnett’s test) or two-way ANOVA with multiple comparisons (Dunnett’s test) were employed. A p-value of less than 0.05 was considered statistically significant.

## Results

### PD-L1 expression is increased in mouse bladder tumor tissue compared to normal bladder tissue

**Fig. 1:**
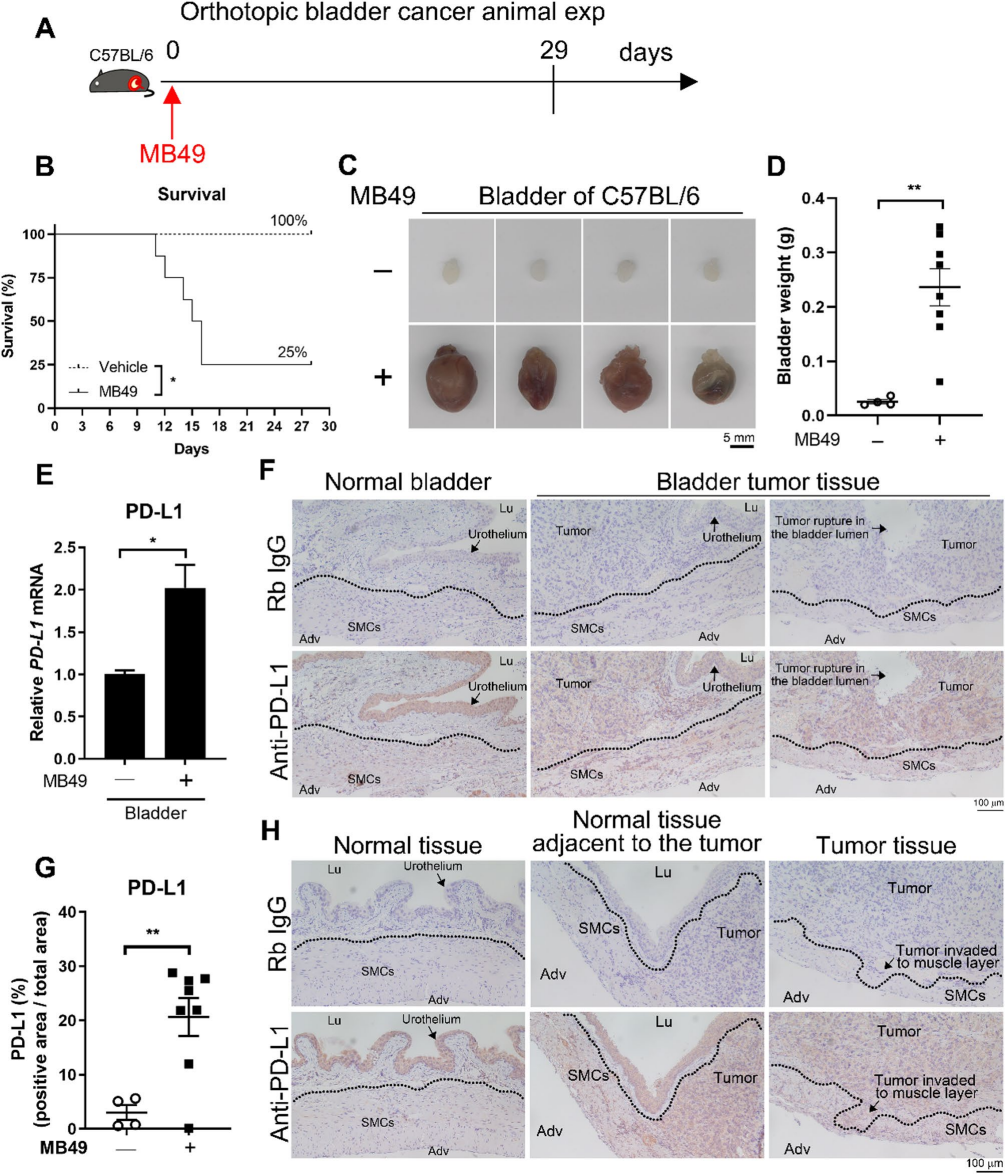
The expression of PD-L1 is upregulated in bladder with tumor. C57BL/6 mice were intravesically implanted with (*n* = 8) or without (*n* = 4) 6 × 10^4^ MB49 mouse bladder tumor cells. The mice were euthanized at the humane or experiment endpoint, and the bladders were collected and weighed. **A** Schematic representation of the experimental timeline for the animal study. **B** The survival of the mice was monitored daily and analyzed using the log-rank (Mantel-Cox) test. **C** The bladder was collected at the endpoint (Day 29) or a humane endpoint and photographed by using an Olympus SZX7 microscope. Representative images of the bladder from control and treated mice are shown. Scale bar represents 5 mm. **D** Weight of the bladder and the tumor within it from treated mice. Data represent mean ± SEM. **E** PD-L1 gene expression was analyzed by qPCR. **F** The PD-L1 levels in bladder tissues were assessed by immunohistochemistry. Representative images of the bladder from vehicle- or MB49-treated mice. The scale bar represents 100 μm. **G** PD-L1 immunopositive areas in paraffin-embedded bladder tissues were quantified using ImageJ software, expressed as a percentage of total bladder area in each section. **H** Representative magnified images of the bladder from mice treated with either vehicle or MB49 cells. Normal bladder (without MB49 implantation) tissue is elastic, with rugae in the urothelium and a relatively thick smooth muscle layer. In bladder tumors, distension may cause urothelial flattening and thinning of the smooth muscle. With progression, tumors can invade and infiltrate the smooth muscle layer. The scale bar corresponds to 100 μm. Data represent mean ± SEM. * *P* < 0.05; ** *P* < 0.01

Numerous studies indicate that PD-L1 expression in bladder cancer patients is associated with an unfavorable prognosis [31, 32]. PD-L1 expression may lead to a decrease in cancer immunosurveillance or responsiveness to therapy, resulting in treatment failure and cancer progression [33]. We aimed to determine whether PD-L1 is upregulated in an orthotopic mouse model of bladder cancer. If upregulation is confirmed, we can utilize this model to evaluate the efficacy of metformin in reducing PD-L1 expression in bladder cancer. We implanted MB49 mouse bladder cancer cells into immunocompetent C57BL/6 mice to establish a syngeneic orthotopic bladder cancer animal model (Fig. 1A). Survival analysis revealed that mice implanted with MB49 tumor cells had significantly reduced survival compared to mice treated with vehicle (Fig. 1B). Additionally, the implanted MB49 cancer cells developed into tumors, causing increases in both the size and weight of the bladder (Fig. 1C and D). The gene expression of PD-L1 was significantly increased in bladder tumor tissue compared to normal bladder (Fig. 1E). Likewise, immunohistochemical staining confirmed that PD-L1 protein expression was significantly increased in bladder tumor tissue compared to normal bladder tissue (Fig. 1F and G). Moreover, in normal bladder tissue, PD-L1 protein was primarily expressed in the urothelium; however, in tumor-bearing mice, elevated PD-L1 protein expression occurred not only in the tumor, but also in the adjacent smooth muscle (Fig. 1H). These data indicate that PD-L1 expression is upregulated in the orthotopic bladder cancer mouse model.

### Metformin reduces PD-L1 gene and protein expression in the mouse bladder cancer cell line MB49 and suppresses bladder cancer cell proliferation in vitro

**Fig. 2:**
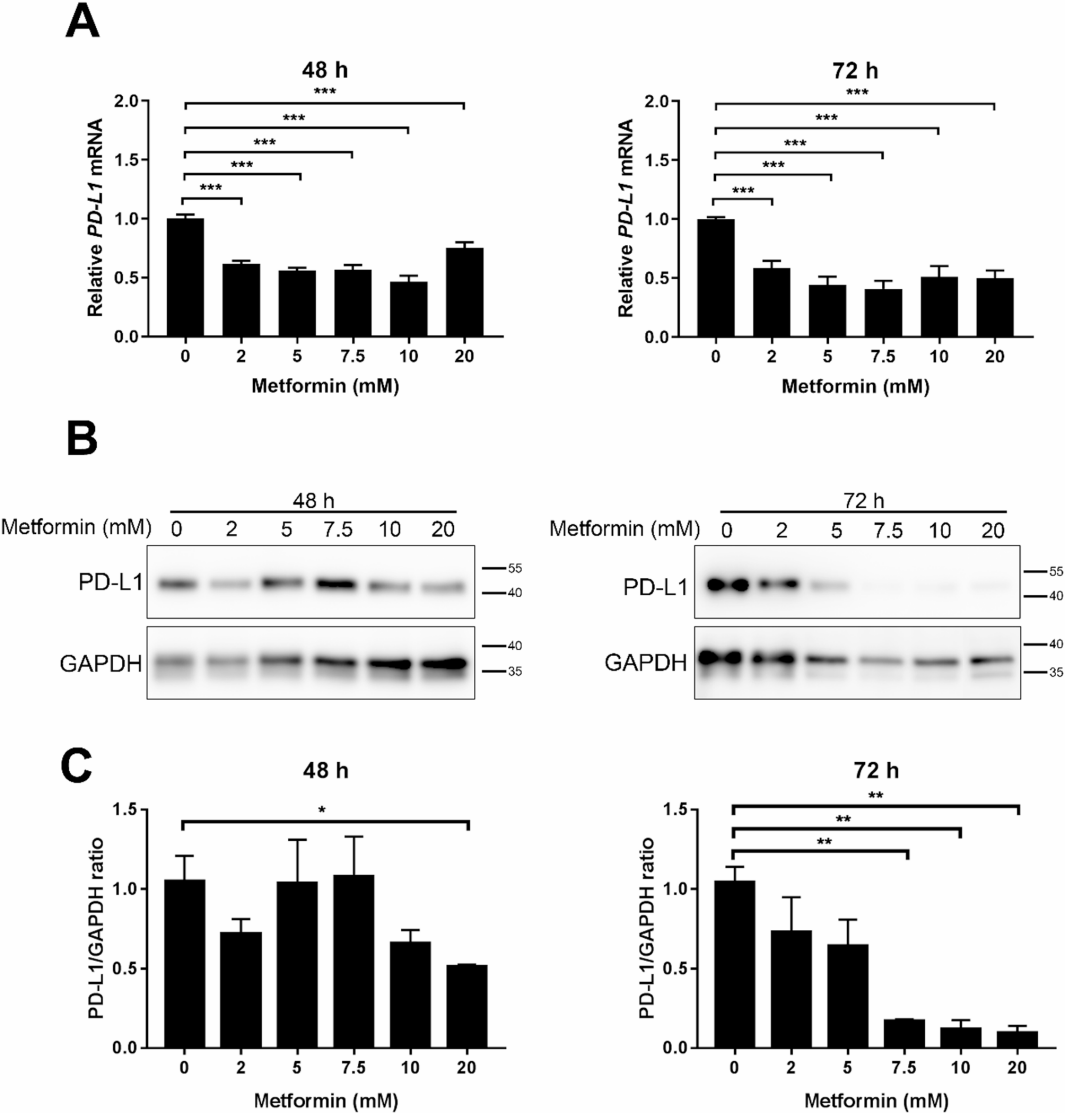
Metformin reduces both PD-L1 gene and protein expression in the mouse bladder cancer cell line MB49 *in vitro*. MB49 cells were treated with the indicated concentrations of metformin for 48–72 h. **A** PD-L1 gene expression was analyzed by qPCR. **B** Cell lysates were immunoblotted with antibodies against PD-L1 and GAPDH. **C** Densitometric analysis of PD-L1 protein expression was performed relative to GAPDH levels. All experiments were repeated at least in duplicate with similar results. * *P* < 0.05; ** *P* < 0.01; *** *p* < 0.001

Next, we treated MB49 mouse bladder cancer cells *in vitro* with metformin for either 48–72 h. RNA and protein were then extracted from the treated cells to assess PD-L1 gene and protein expression. Analysis of PD-L1 gene expression showed that metformin caused a reduction in PD-L1 expression as the concentration of metformin increased, both at 48 h and 72 h (Fig. 2A). Immunoblot analysis confirmed that metformin reduced PD-L1 protein expression for both treatment durations (Fig. 2B-C).

**Fig. 3:**
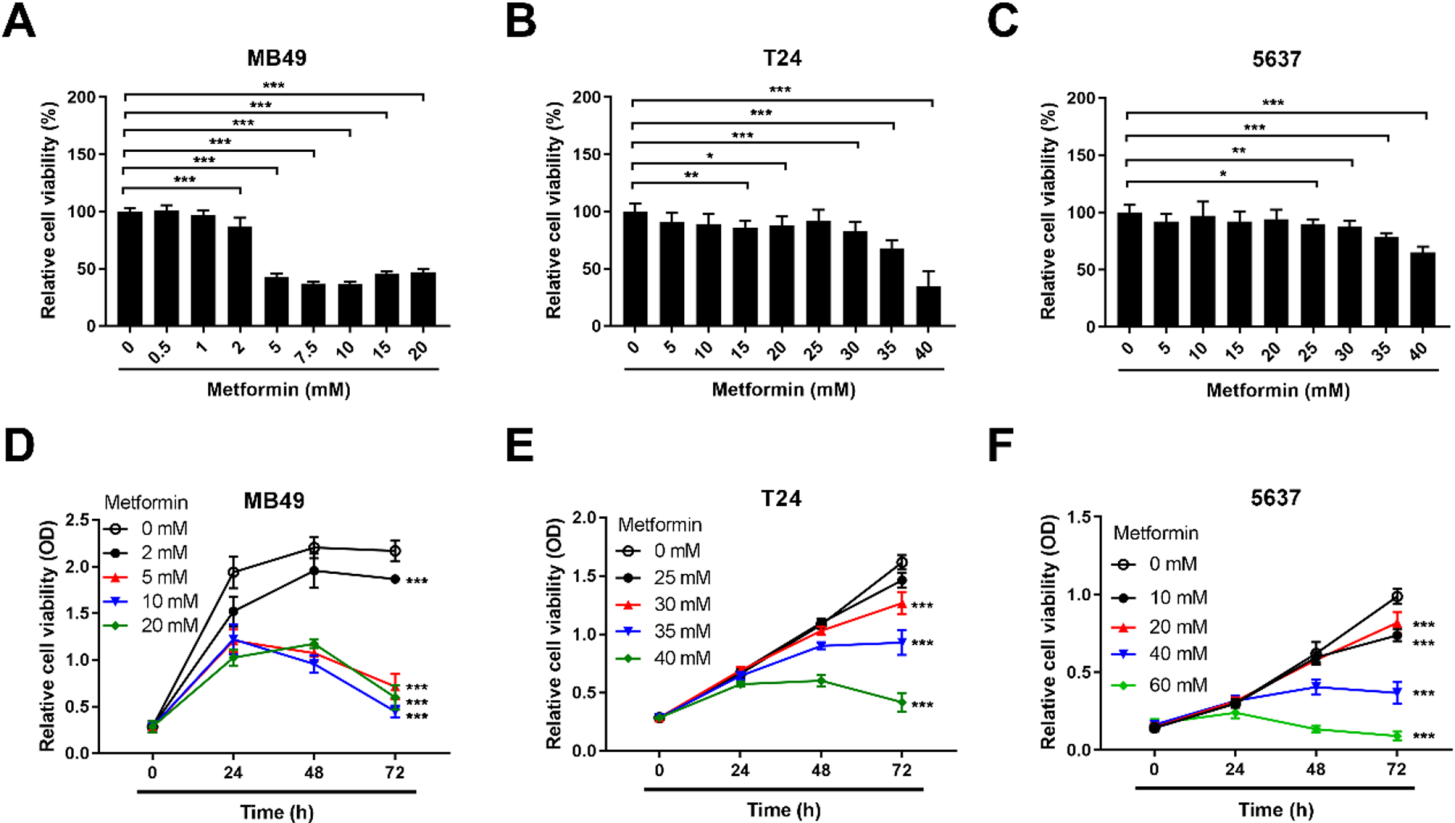
Metformin suppressed bladder cancer cell proliferation in a dose- and time-dependent manner. **A**-**C** Mouse bladder cancer cell line MB49 and two human bladder cancer cell lines T24 and 5637 were treated with a range of concentrations of metformin for 48 h and the cell viabilities were determined using the MTS method. **D**-**F** The effect of different concentrations of metformin over 24, 48, and 72 h on the proliferative ability of MB49, T24, and 5637 cell lines was assessed using MTS assays to measure cell viability. All experiments were repeated at least three times with similar results. Data represent mean ± SEM of three experiments. * *P* < 0.05; ** *P* < 0.01; *** *p* < 0.001

A recent study demonstrated that bladder cancer cell-intrinsic PD-L1 regulates tumor growth and metastatic spread *in vivo* through the modulation of mTOR (mammalian target of rapamycin) activity [34]. Additionally, metformin has been reported to inhibit bladder cancer cell proliferation via the phosphatidylinositol 3-kinase (PI3K)/Ak strain transforming (AKT)/mTOR pathway [35]. Therefore, we assessed the inhibitory effect of metformin on the proliferation of mouse MB49 cells, as well as the human muscle-invasive bladder cancer cell lines T24 and 5637, by using tetrazolium-based assays. The results showed that metformin inhibited the growth of both mouse MB49 and human T24 and 5637 bladder cancer cells in a dose-dependent (Fig. 3A-C) and time-dependent (Fig. 3D-F) manner *in vitro*. These data suggest that metformin can reduce PD-L1 expression in MB49 mouse bladder tumors. In addition to decreasing PD-L1 expression, metformin also suppresses bladder tumor growth. Therefore, the potential of metformin to downregulate PD-L1 expression and promote tumor regression *in vivo* warrants evaluation using an orthotopic bladder cancer mouse model, which we describe below.

### Assessment of the maximum tolerated dose of Metformin in C57BL/6 mice

**Fig. 4:**
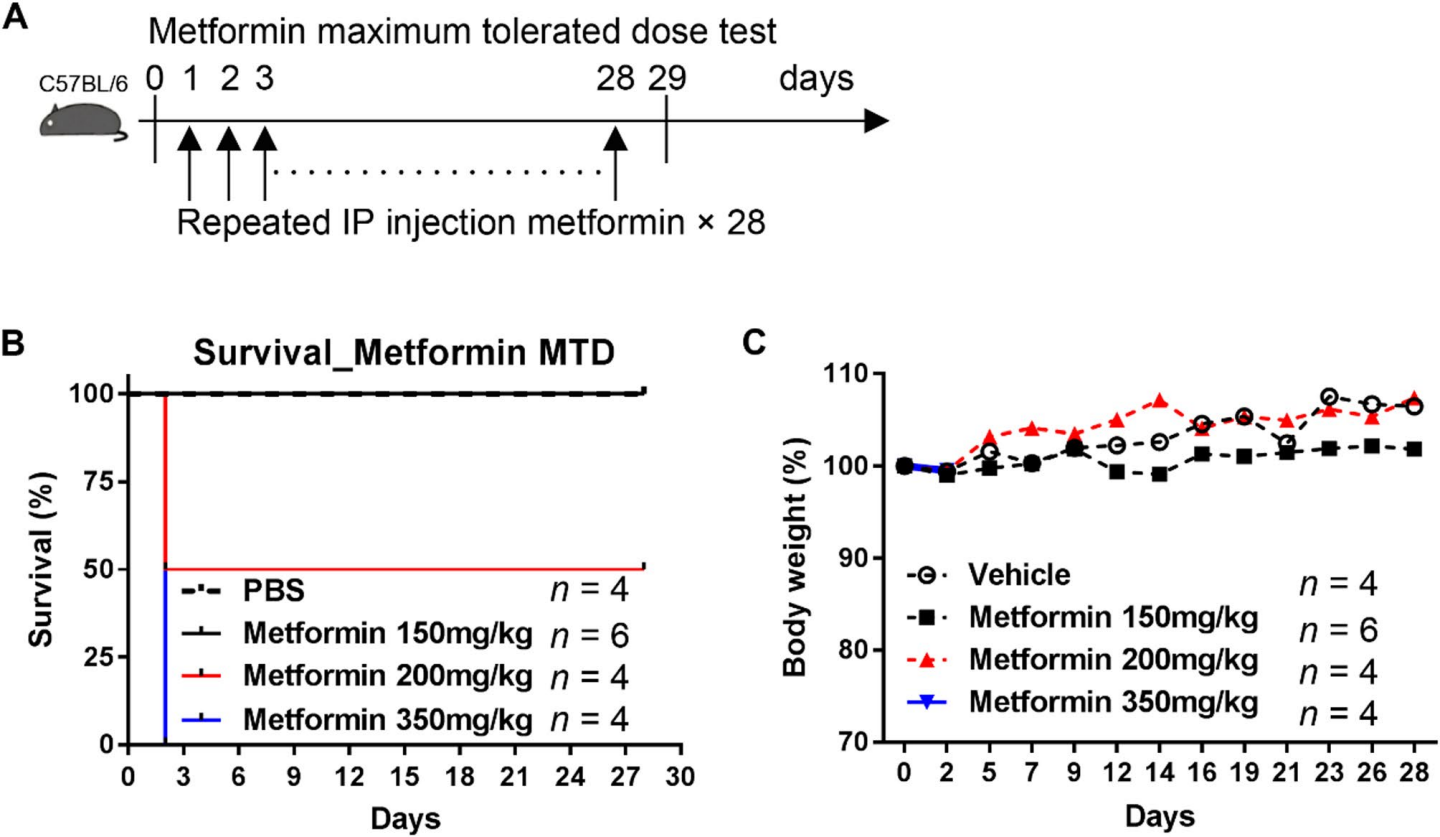
Assessment of the maximum tolerated dose (MTD) of metformin in C57BL/6 mice. C57BL/6 mice were administered PBS, 150, 200, and 350 mg/kg metformin by intraperitoneal injection once daily for 28 days. **A** Schedule of the animal experiments. **B** Mouse survival was monitored daily and analyzed using the log-rank (Mantel-Cox) test. **C** To calculate the change of body weight as a percentage (%), the body weight at Day n was divided by the weight at Day 0 of each mouse and multiplied by 100. The data are expressed as change in percentage (%)

Prior to evaluating the efficacy of metformin in preventing bladder cancer, we first performed an MTD test of metformin in C57BL/6 mice. MTD is defined as the highest dose of a medicine or treatment to which animals are exposed in toxicological studies that produces the desired effect without resulting in unacceptable adverse side effects. The doses of metformin we administered were 150 mg/kg, 200 mg/kg, and 350 mg/kg. Metformin was administered via daily intraperitoneal injection, and the survival of treated mice was monitored daily for 4 weeks (Fig. 4A). Survival analysis indicated that all mice injected with 350 mg/kg of metformin were dead by the following day. Of the mice receiving 200 mg/kg metformin, 50% were dead after one day (Fig. 4B), whereas the remaining mice survived until the end of the experiment without any abnormal signs. Notably, the mice receiving 150 mg/kg of metformin daily showed no abnormal behaviors or deaths (Fig. 4B), and their body weight slightly increased till the experimental endpoint (Fig. 4C). These results suggest that the safe dose of metformin should be less than 200 mg/kg, and that 150 mg/kg is safe and suitable for daily treatment of mice with bladder cancer.

### Metformin effectively prevents bladder cancer in C57BL/6 mice and is associated with downregulation of PD-L1 in bladder tumors

**Fig. 5:**
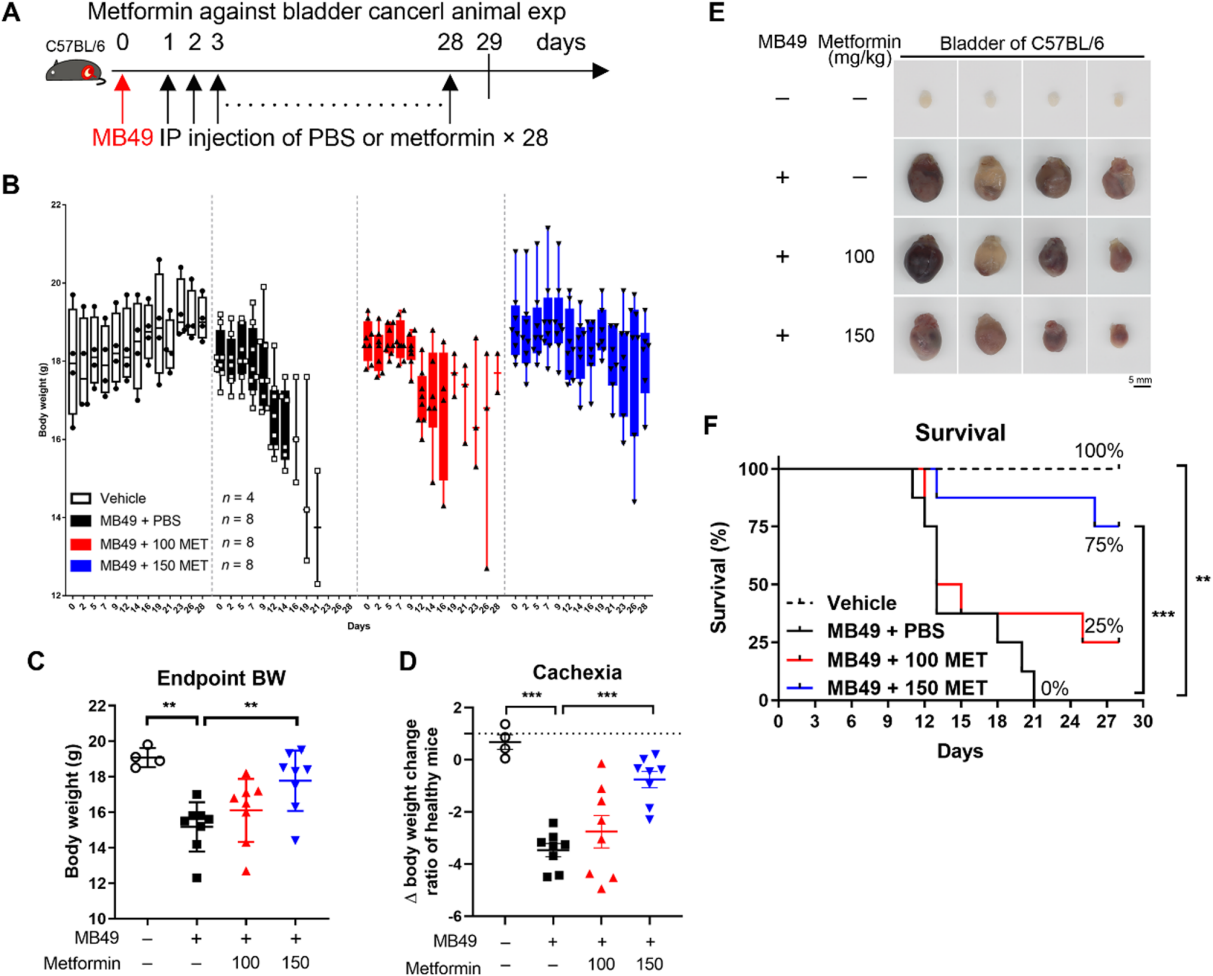
Metformin effectively inhibits bladder cancer progression by attenuating weight loss and improving survival in C57BL/6 mice. C57BL/6 mice were randomly assigned to 4 groups: vehicle (*n* = 4), MB49 + PBS (*n* = 8), MB49 + 100 mg/kg metformin (*n* = 8), and MB49 + 150 mg/kg metformin (*n* = 8). After implantation of 6 × 10^4^ MB49 tumor cells into the bladder, the mice were intraperitoneally injected with PBS or the indicated concentration of metformin the next day and once a day for 28 days. **A** Schedule of the animal experiments. **B** The body weight was recorded at Day 0 and 1 day after the first intraperitoneal injection and then three times a week for 4 weeks. **C** The body weight of treated mice at the humane or experimental endpoint. **D** The delta body weight change ratio was calculated to assess cancer-induced cachexia. This was determined by dividing the body weight change of tumor-bearing mice (excluding bladder/tumor weight) by that of healthy control mice (excluding bladder weight). The formula is as follows: Δ body weight change ratio = [(BW_Day n − bladder/tumor weight) − BW_Day 0]_tumor mice/[(BW_Day n − bladder weight) − BW_Day 0]_healthy mice, where BW denotes body weight. **E** The bladder was collected at the experimental endpoint (Day 29) or a Humane endpoint and photographed. Representative images of the bladder from treated mice. Scale bar represents 5 mm. **F** Mouse survival was monitored daily and analyzed using the log-rank (Mantel-Cox) test. 100 MET: 100 mg/kg metformin; 150 MET: 150 mg/kg metformin, ** *P* < 0.01; *** *p* < 0.001

We next evaluated the prophylactic antitumor efficacy of metformin in a syngeneic orthotopic bladder cancer mouse model, using PD-L1-positive MB49 bladder cancer cells in C57BL/6 mice. Metformin was administered at doses of 0 (PBS), 100, and 150 mg/kg daily via intraperitoneal injection until Day 28, starting after intravesical implantation of the tumor (Fig. 5A). Analysis of the body weight data showed that the weights of vehicle-treated mice (receiving intraperitoneal injections of PBS without tumor implantation) increased steadily, whereas the body weight of MB49 + PBS-treated mice decreased rapidly 2 weeks post-tumor implantation (Fig. 5B). Intraperitoneal injections of 100 mg/kg metformin slightly alleviated weight loss caused by MB49 tumor cells, while treatment with 150 mg/kg metformin led to a more pronounced alleviation (Fig. 5B). The endpoint body weight data show that MB49 + PBS-treated mice lost approximately 4 g compared to vehicle-treated mice (Fig. 5C). The intraperitoneal injections of 150 mg/kg metformin significantly mitigated the weight loss caused by MB49 tumor cells at the experimental endpoint or humane endpoint (Fig. 5C). Weight loss greater than 5% over a period of 6 months is a primary sign of cancer cachexia, a secondary condition in cancer patients that leads to progressive dysfunction. Cachexia is characterized by a systemic inflammatory response, reduced food intake, and abnormal metabolism [36]. It accounts for 20% of all cancer-related deaths and is associated with poor prognosis [37]. To assess cancer cachexia induced by MB49 tumor cells, we calculated the ratio of the change in body weight of the tumor group (endpoint body weight on Day n without bladder (tumor weight) minus initial body weight on Day 0) to the change in body weight of healthy mice (endpoint body weight without bladder minus initial body weight on Day 0). The delta body weight Change ratio showed that the weight Change in vehicle-treated mice was comparable to that of healthy mice, resulting in a ratio close to 1 (Fig. 5D). In contrast, mice treated with MB49 tumor cells experienced weight loss approximately 2.5 to 3 times greater than the weight gain observed in healthy mice. The delta body weight change ratio confirmed significant weight loss in mice treated with MB49 tumor cells compared to vehicle-treated mice (Fig. 5D). Treatment with 150 mg/kg metformin significantly mitigated MB49 tumor cell-induced cancer cachexia compared to treatment with PBS (Fig. 5D). There was a trend toward a decrease in size (Fig. 5E and Figure S1A) and weight (Figure S1B) of the bladder tumor with increasing doses of metformin, although this change was not statistically significant. Notably, this downward trend was associated with improved survival (Fig. 5F). Survival analysis revealed that all mice treated with MB49 + PBS died, whereas treatment with 150 mg/kg metformin significantly reduced the mortality induced by MB49 tumor cells compared to MB49 + PBS-treated mice (Fig. 5F).

**Fig. 6:**
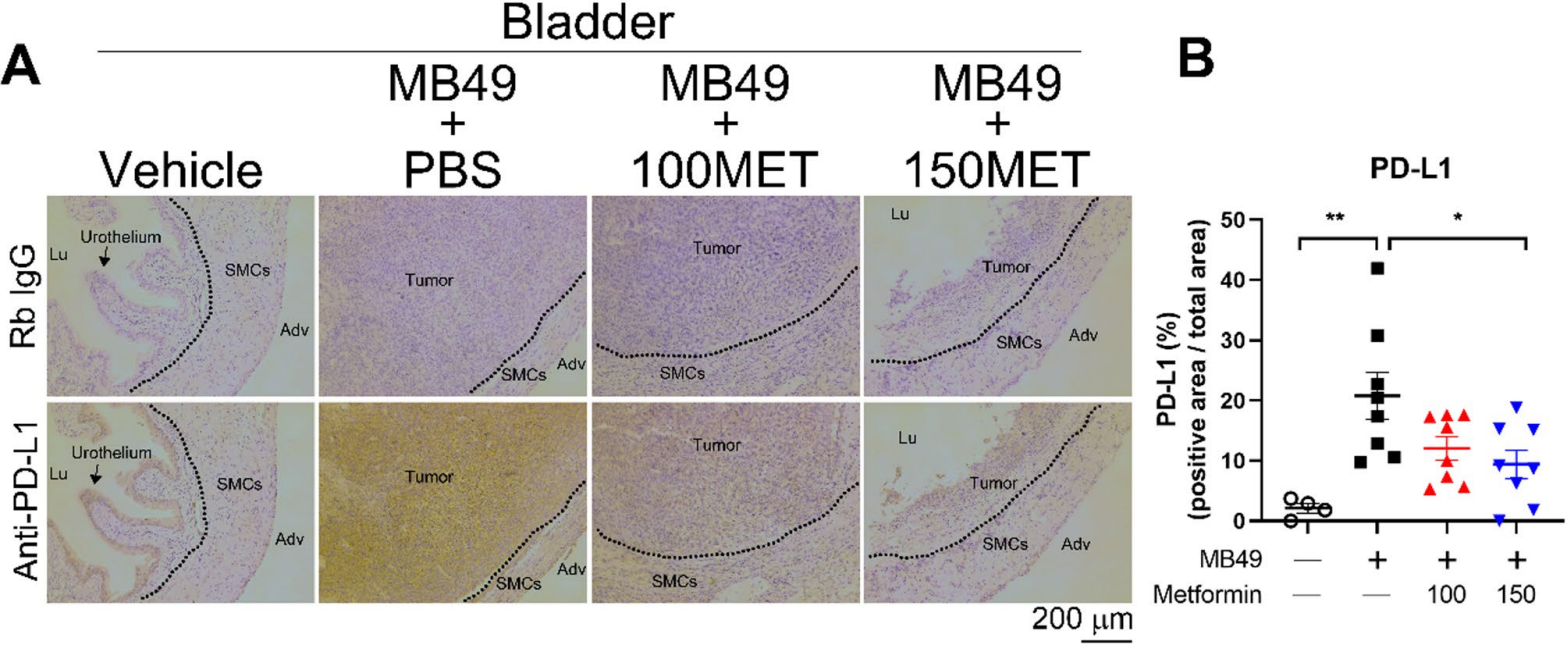
Metformin downregulated PD-L1 expression in mouse bladder tumors. C57BL/6 mice were assigned to the following 4 groups: Vehicle (PBS, *n* = 4), MB49 + PBS (*n* = 8), MB49 + 100 mg/kg metformin (*n* = 8), and MB49 + 150 mg/kg metformin (*n* = 8). Following the implantation of MB49 tumor cells (6 × 10⁴ cells) into the bladder, the mice were intraperitoneally injected with PBS or the indicated concentrations of metformin the following day, and once daily for 28 days. At the humane and experimental endpoints, the bladders of treated mice were collected, paraffin-embedded, and sectioned for immunohistochemical analysis of PD-L1 expression in the tissue. **A** Sections from treated mice were stained immunohistochemically for PD-L1 in bladder tumor tissue. The representative images show bladder sections treated with vehicle, MB49 + PBS, MB49 + 100 mg/kg metformin, and MB49 + 150 mg/kg metformin. Immunopositive areas in paraffin-embedded bladder tissues of treated mice were quantified using ImageJ software as a percentage of total bladder area in each section. The scale bar represents 200 μm. **B** Quantification of PD-L1 by ImageJ software. 100 MET: 100 mg/kg metformin; 150 MET: 150 mg/kg metformin, * *p* < 0.05, ** *p* < 0.01

We hypothesized that the alleviation in MB49-induced cachexia and the prolonged survival observed with metformin treatment were associated with downregulation of PD-L1 expression. To investigate this, PD-L1 protein levels in tumor tissue were assessed using immunohistochemistry. Immunostaining analysis revealed a significant increase in PD-L1 expression in bladder from MB49 + PBS-treated mice compared to vehicle-treated controls (Fig. 6A). Furthermore, metformin treatment at 150 mg/kg significantly reduced the elevated PD-L1 expression in bladder tumor tissue (Fig. 6A and B). Collectively, these results suggest that the increased expression of PD-L1 during tumor progression can be reversed by metformin, and that this downregulation of PD-L1 is associated with a reduction in bladder tumor-induced cachexia and improved survival. Therefore, we propose that metformin may serve as an adjunctive therapy to prevent the progression and recurrence of bladder tumors, offering a potential therapeutic approach for the prevention of bladder cancer.

## Discussion

With the growing understanding of immune checkpoint mechanisms and the success of using antibodies to block these checkpoints in cancer treatment, immunotherapy has emerged as one of the most promising areas in cancer research, offering the potential for durable therapeutic effects. However, only a subset of cancers currently responds favorably to checkpoint inhibition. A deeper understanding of the mechanisms underlying the cancer response and resistance is crucial for fully harnessing the potential of immunotherapy to treat the majority of cancers. Bladder cancer, a prevalent and aggressive malignancy, has been successfully managed in both early and advanced stages through various immunotherapeutic strategies, including intravesical BCG instillation and PD-1/PD-L1 checkpoint blockade therapies [3, 38]. Thus, bladder cancer serves as an excellent model for studying cancer immune response mechanisms and improving the efficacy of immunotherapy. Moreover, bladder cancer is an ideal tumor model for testing and applying cancer prevention strategies; however, there are limited studies on the use of metformin in the management of bladder cancer. In the current study, we demonstrated elevated PD-L1 expression in an orthotopic bladder cancer animal model, at both the mRNA and protein levels. *In vitro* experiments showed that metformin reduced both the gene and protein expression of PD-L1. Furthermore, metformin not only inhibited bladder cancer cell growth *in vitro* but also decreased PD-L1 expression *in vivo*, leading to tumor shrinkage and improved survival rates in mice.

Previous study has shown that genetic deletion or antibody-mediated neutralization of PD-L1 in bladder tumor cells can suppress *in vivo* tumor growth and metastatic potential even in immunodeficient models, suggesting a tumor-intrinsic role for PD-L1 in promoting oncogenic signaling [34]. According to previous literature, urothelial exposure to metformin via urine may be significantly higher than exposure via systemic circulation. Following oral administration of metformin in both humans and murine models, plasma concentrations typically reach only micromolar (µM) levels, while *in vitro* studies have shown that millimolar (mM) concentrations are required to inhibit bladder cancer cell proliferation and mTOR signaling [35, 39, 40], suggesting plasma levels may be insufficient to exert a direct inhibitory effect on mTOR phosphorylation. Importantly, prior work has reported that urinary metformin concentrations in mice were approximately 5–8 mM, which is higher than plasma levels (typically 20–30 µM) [40]. This urinary concentration is comparable to the effective dose observed in *in vitro* models for inhibiting bladder cancer cell growth and mTOR signaling. These findings suggest that tumor-intrinsic PD-L1 promotes tumor growth by enhancing mTOR signaling and AKT phosphorylation [34]. Based on these observations and our own results, we propose that the anti-tumor effect of metformin is primarily mediated through the inhibition of tumor-intrinsic growth pathways, with only a limited contribution from immune-mediated mechanisms. Nevertheless, the potential immunomodulatory effects of metformin, particularly its impact on tumor-infiltrating lymphocytes and the tumor immune microenvironment, remain an important area for future investigation.

Intravesical administration of metformin more closely mimics clinical intravesical therapy for bladder cancer. Previous studies have successfully applied this approach in murine bladder cancer models [39, 41]. However, this method is limited by the duration of anesthesia in mice, and the drug often leaks out after catheter removal, making the procedure labor-intensive and time-consuming. Due to the invasive nature of intravesical instillation and its dependence on anesthesia, frequent administration is not feasible, thus requiring higher drug doses. Nevertheless, the localized delivery reduces concerns regarding systemic side effects such as metformin-induced lactic acidosis. In our study, we employed intraperitoneal injection of metformin, which offers a more practical and time-efficient method, ensuring consistent dosing. The administered dose was below the maximum tolerated dose in mice, maintaining safety and minimizing the risk of lactic acidosis. Moreover, systemic administration better simulates the pharmacokinetics of oral metformin absorption in humans. Future studies evaluating safe oral dosing regimens may further improve treatment convenience and reduce the clinical burden on both patients and healthcare providers.

Several studies have shown that PD-L1 expression in bladder cancer patients is associated with stage progression and poor prognosis [14–16]. The interaction between PD-L1 and PD-1 on T cells promotes T cell apoptosis or anergy, which can impair T cell-mediated immune responses. This may contribute to reduced cancer immunosurveillance or a diminished response to therapy, ultimately leading to treatment failure and cancer progression [42]. Moreover, an increase in PD-L1 expression was observed in patients with BCG-resistant NMIBC following intravesical BCG treatment, which may contribute to the immune escape mechanism and poor responses to BCG therapy [43–45]. A recent study indicates that tumors in patients with NMIBC who do not respond to BCG therapy exhibit elevated expression of PD-L1, thereby neutralizing the cytotoxicity of co-localized CD8+ cells [44]. In addition to the interaction of PD-L1/PD-1 in suppressing T cell function and facilitating immune evasion in cancer, PD-L1 on cancer cells can also transmit signals that drive tumor progression. For instance, PD-L1 activation can trigger the PI3K-AKT-mTOR and MAPK signaling pathways, which in turn promote processes such as proliferation, the epithelial-to-mesenchymal transition, chemoresistance, and the acquisition of stem cell-like properties [46–48]. This pro-oncogenic phenomenon is also observed in bladder cancer, where bladder cancer cell-intrinsic PD-L1 enhances mTOR signaling, promoting immune-independent cell growth and metastasis, consistent with findings in other malignancies [34]. Treatment with anti-PD-L1 antibodies or PD-L1 knockout reduces bladder cancer cell proliferation and growth, indicating direct signaling effects [34]. The role of PD-L1 in bladder cancer also includes mediating resistance to commonly used chemotherapy agents, such as cisplatin and gemcitabine, as well as to the mTOR inhibitor rapamycin [34]. Therefore, bladder cancer cell-intrinsic PD-L1 signaling modulates critical pathways involved in virulence and treatment resistance, indicating potential novel and actionable treatment targets.

Recent evidence supports the potential of metformin as an adjunct to immunotherapy in cancer treatment, particularly due to its effects on PD-L1 expression, T cell function, and modulation of the immunosuppressive tumor microenvironment [26–28]. In our study, we demonstrated that metformin significantly suppresses PD-L1 expression in a preclinical model of bladder cancer, which may alleviate immune evasion and promote more effective antitumor immune responses. Tumor cell death induced by metformin can also reinforce the expansion of tumor-infiltrating lymphocytes (TILs) [28], thereby enhancing immunosurveillance and contributing to improved therapeutic outcomes. Several preclinical studies have shown that combining metformin with immune checkpoint blockade or targeted therapies results in synergistic antitumor effects [49, 50]. These findings suggest that metformin may help overcome resistance to immune checkpoint inhibitors (ICIs), which remains a major clinical challenge due to the dependency of ICI efficacy on pre-existing endogenous immune responses [50].

Mechanistically, metformin inhibits bladder cancer cell proliferation through induction of cell cycle arrest and suppression of key oncogenic pathways such as PI3K/AKT/mTOR and STAT3, which are critical regulators of both tumor growth and PD-L1 expression [35, 40, 51–54]. Clinically, ICIs may enhance the efficacy of targeted therapy by impacting molecules that regulate both PD-L1 expression and cell proliferation [55]. Although metformin monotherapy shows limited efficacy in highly aggressive tumors, preclinical studies have demonstrated that its combination with PD-1/PD-L1 blockade improves intratumoral T-cell responses and promotes tumor regression [49]. By concurrently targeting immune evasion and tumor proliferation, metformin may enhance the clinical efficacy of PD-1/PD-L1 blockade while also contributing to a reduction in treatment costs due to its established safety profile and affordability. Clinical observations have reported favorable trends in treatment outcomes with this combination [56, 57], although not all studies reached statistical significance. These findings highlight the translational potential of metformin as a combinatorial agent with ICIs, particularly in bladder cancer, and support further investigation in clinical trials.

Metformin exerts its antitumor effects through multiple mechanisms. In addition to directly activating AMP-activated protein kinase (AMPK), which inhibits the PI3K/AKT/mTOR signaling pathway [35, 40, 54], metformin inhibits the mRNA translation of sterol regulatory element-binding protein 1c (SREBP-1c) and fatty acid synthase (FASN), thereby suppressing lipid synthesis through the targeting of clusterin in bladder cancer [41]. Metformin has also been shown to indirectly attenuate cancer cell growth by systemically reducing glucose and insulin metabolism [22]. Recent studies further demonstrate that metformin modulates the differentiation and activity of T cells [28, 58], suggesting that its antitumor effects may be tied to immune responses. Our findings support this hypothesis by linking metformin to both downregulation of PD-L1 expression and suppression of tumor cell growth in bladder cancer.

Although blocking the PD-1/PD-L1 axis with ICIs has shown great benefit in BCG-unresponsive NMIBC or advanced bladder cancer, almost all PD-1/PD-L1 blockades have been reported to be associated with immune-related adverse events (IRAEs), the incidence of which may reach as high as 90% with single-agent ICI therapy, including fatigue, anorexia, diarrhea, hypothyroidism, overactive thyroid, pneumonitis, skin rash, and colitis [19, 59]. Apart from the non-specific activation of the immune system, off-target binding is another significant cause of antibody-induced adverse events. These antibodies may exhibit cross-reactivity to structurally similar targets with distinct functions, resulting in unexpected side effects. IRAEs are typically well-managed with symptomatic treatment; however, severe and fatal adverse events may still occur [60]. Furthermore, the response rate to ICIs is low for bladder cancer and imposes a high-cost burden for patients [18, 21]. Metformin has been employed in clinical practice for decades and has consistently demonstrated good safety and tolerability profiles [61]. Common side effects, such as nausea, gastrointestinal discomfort, or diarrhea, are typically mild. More serious adverse events, such as severe allergic reactions or lactic acidosis, are rare. The risk of lactic acidosis is elevated in individuals with significant renal impairment, leading clinicians to generally avoid prescribing metformin in such cases [61]. Otherwise, metformin is cost-effective and its antitumor effects are well-documented.

Our data demonstrate the use of a bladder cancer animal model to evaluate the role of metformin in preventing tumor progression, showing it to be both safe and effective. We also identified PD-L1 expression in the mouse bladder cancer model and highlighted the inhibition of PD-L1 expression by metformin, which alleviated MB49-induced cachexia and improved mouse survival. This research may inform the development of metformin as a prophylactic anticancer agent for bladder cancer and other malignancies.

## Conclusions

Elevated PD-L1 expression in patients with bladder cancer has been associated with cancer progression and unfavorable prognosis. It could impair cancer immunosurveillance or reduce the effectiveness of therapy, potentially leading to treatment failure. In this study, our results demonstrate increased PD-L1 expression in the development of bladder cancer in a syngeneic orthotopic bladder cancer mouse model. Our results also confirm the prophylactic antitumor efficacy of metformin in both *in vitro* and *in vivo* experiments, which inhibited PD-L1 expression and suppressed bladder cancer cell proliferation, thereby shrinking tumor growth and prolonging mouse survival.

## Supplementary Information

Supplementary Material 1.

Supplementary Material 2.

## Data Availability

Data is provided within the manuscript or supplementary information files.

## Abbreviations

PD-L1: Programmed death ligand 1
PD-1: Programmed death protein 1
MTD: Maximum tolerated dose
IHC: Immunohistochemistry
NMIBC: Non-muscle invasive bladder cancer
MIBC: Muscle-invasive bladder cancer
TURBT: Transurethral resection of bladder tumor
BCG: Bacillus Calmette–Guérin
ICIs: Immune checkpoint inhibitors
GAGs: Glycosaminoglycans
PLL: Poly L-lysine
QPCR: Quantitative polymerase chain reaction
PVDF: Polyvinylidene difluoride
ANOVA: Analysis of variance
PI3K: Phosphatidylinositol 3-kinases
AKT: Ak strain transforming
mTOR: Mammalian target of rapamycin
MAPK: Mitogen-activated protein kinases
STAT3: Signal transducer and activator of transcription 3
AMPK: AMP-activated protein kinase
SREBP-1c: Sterol regulatory element-binding protein 1c
FASN: Fatty acid synthase
IRAEs: Immune-related adverse events
