# Supplementary figures and images for: Metformin as an anti-cancer agent against bladder cancer acts via PD-L1 downregulation in an orthotopic mouse model
Source: BMC Cancer. 2025 Oct 8;25:1534. doi: 10.1186/s12885-025-14930-2 (PMC12506300; doi:10.1186/s12885-025-14930-2)

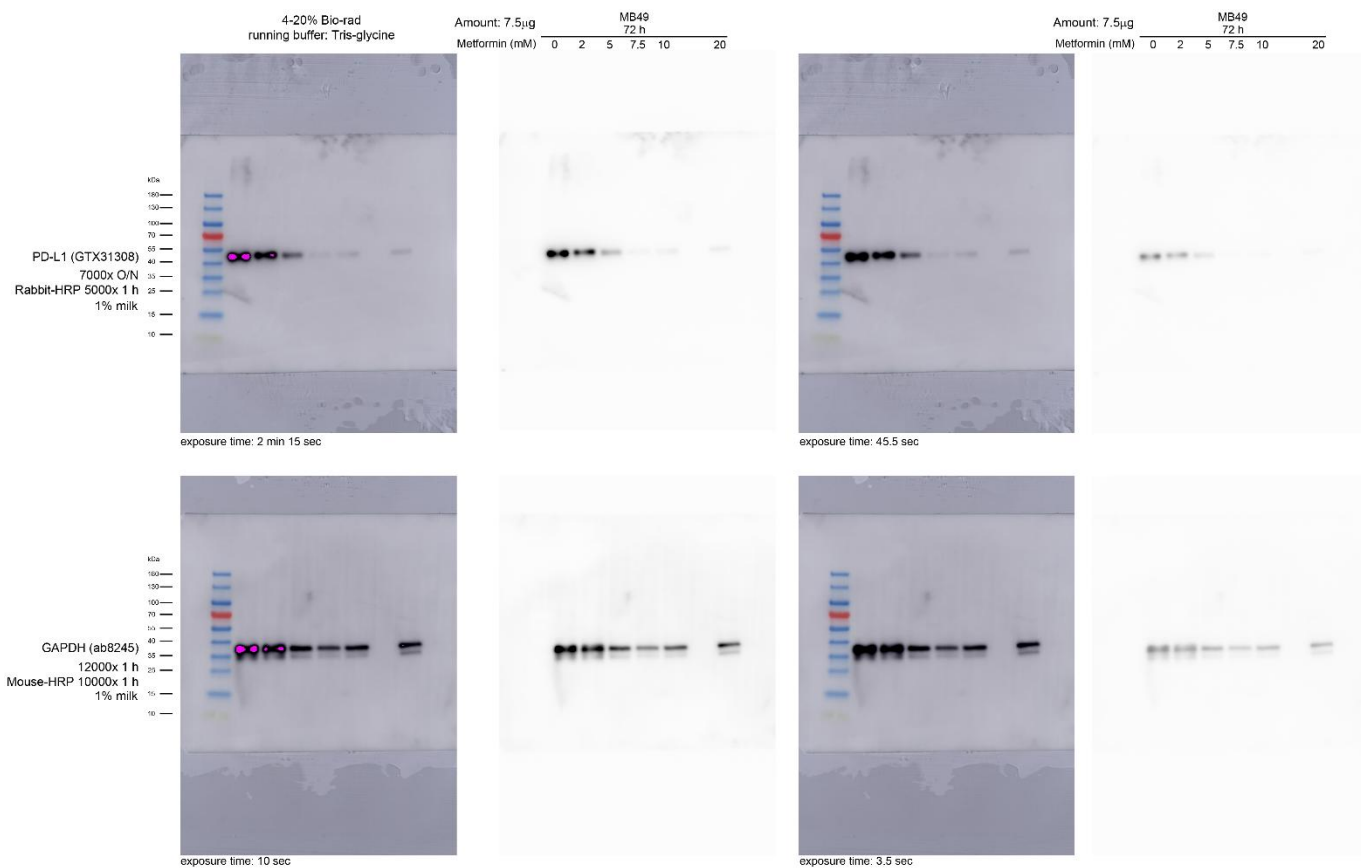

Supplement: Supplementary file 1 — Supplementary Material 1. [file 12885_2025_14930_MOESM1_ESM.pdf]

Fig. 2B original data

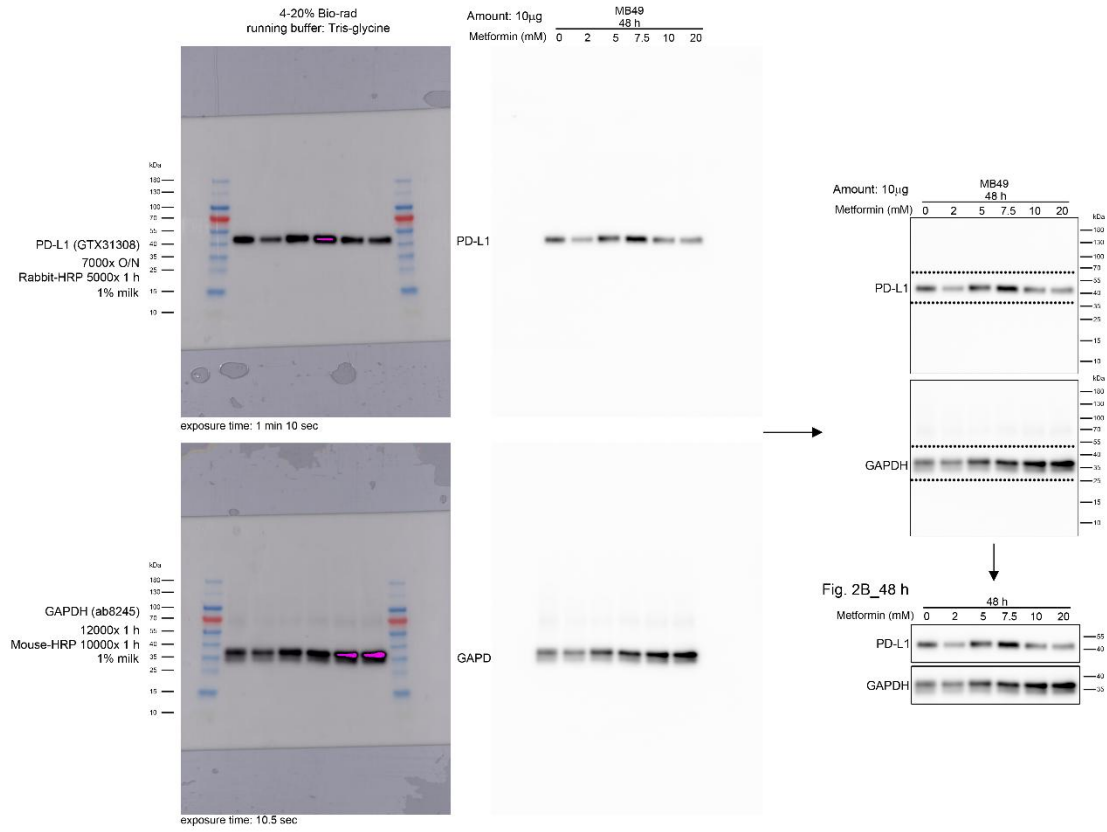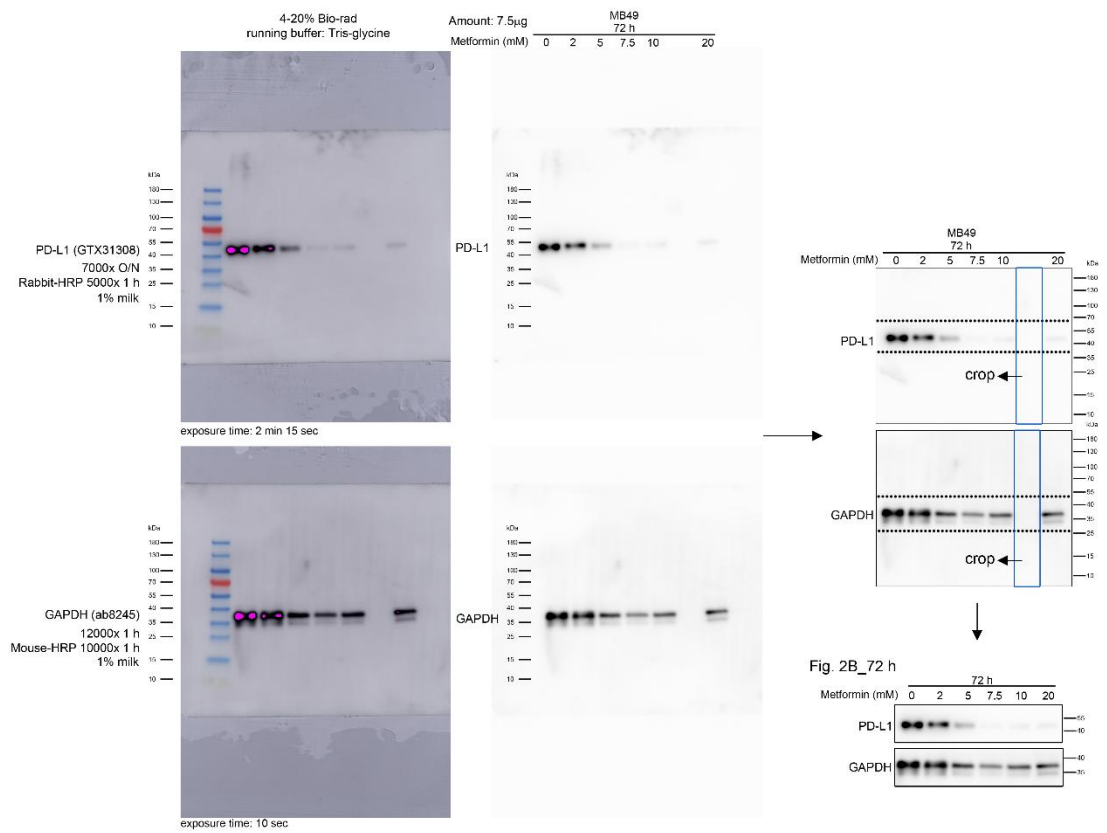

Supplement: Supplementary file 2 — Supplementary Material 2. [file 12885_2025_14930_MOESM2_ESM.pdf]
